# Abdominal Wall Schwannoma: Case Report and Review of the Literature

**DOI:** 10.1155/2013/456863

**Published:** 2013-06-09

**Authors:** A. Mishra, M. Hamadto, M. Azzabi, M. Elfagieh

**Affiliations:** ^1^Department of Radiology, National Cancer Institute, Misrata, Libya; ^2^Department of Surgery, Central Hospital, Tripoli, Libya; ^3^Department of Surgical Oncology, National Cancer Institute, Misrata, Libya

## Abstract

A 29-year-old female had presented to surgical outpatient's department complaining of lump in the anterior abdominal wall. Ultrasound and magnetic resonance imaging revealed a solid degenerated tumor in the anterior abdominal wall. It was surgically excised, and histopathology confirmed it to be “ancient” schwannoma. To our knowledge, this is the second reported case of an abdominal wall ancient schwannoma in the medical literature.

## 1. Introduction 

Schwannoma or neurilemmoma is uncommon tumor that arises from nerve sheath, and most frequently affects the extremities, trunk, heads and neck areas. These tumors are completely benign and are very often an incidental finding. Their presentation in the abdominal wall is extremely rare and we came across only very few reports [1] in medical research database, and to our knowledge, this is the second reported such case. 

## 2. Case Report

A 29-year-old young woman presented to the surgical out patient's department complaining of a painless lump in the left upper abdomen 10 months ago. She also stated that the lump was gradually increasing in size. There was no history of trauma, anorexia, weight loss, or paresthesia. There was no family history of the similar complaint. 

On physical examination, the lump measured 6 cm transversely and was firm, nontender, and sharply demarcated. It was not fixed to the skin of the anterior abdominal wall. On contracting the muscles of the anterior abdominal wall, it became less apparent and associated with restriction of movement. All routine laboratory tests were within normal limits. 

**Figure 1:**
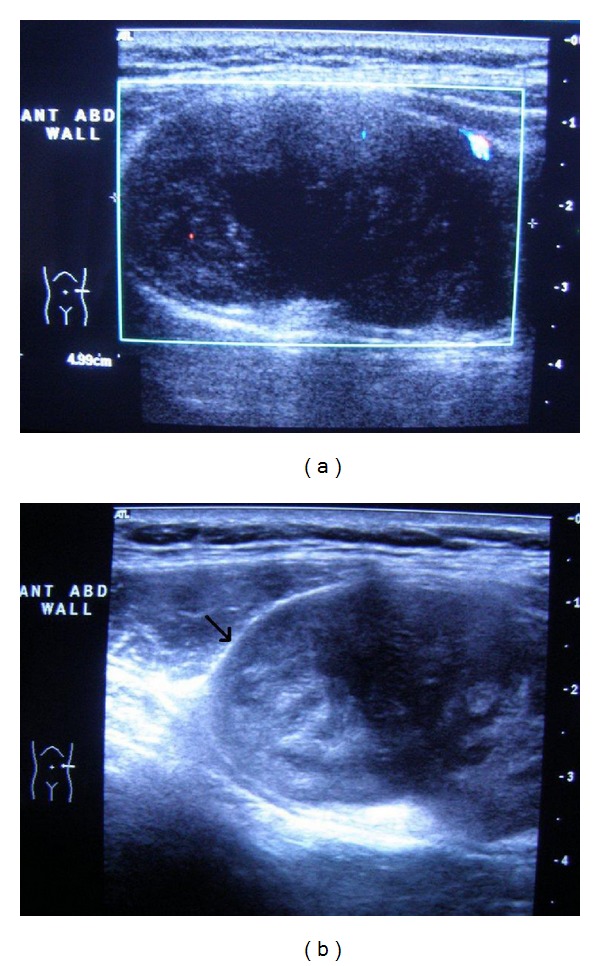
(a) Color flow showing hypovascularity of the mass. (b) B-mode ultrasound shows the well-encapsulated mass in anterior abdominal wall (arrow).

On ultrasound, the mass was encapsulated, solid, flat, and heterogeneous and was located in the anterior abdominal wall. It was hypovascular on color flow (Figures 1(a) and 1(b)).

**Figure 2:**
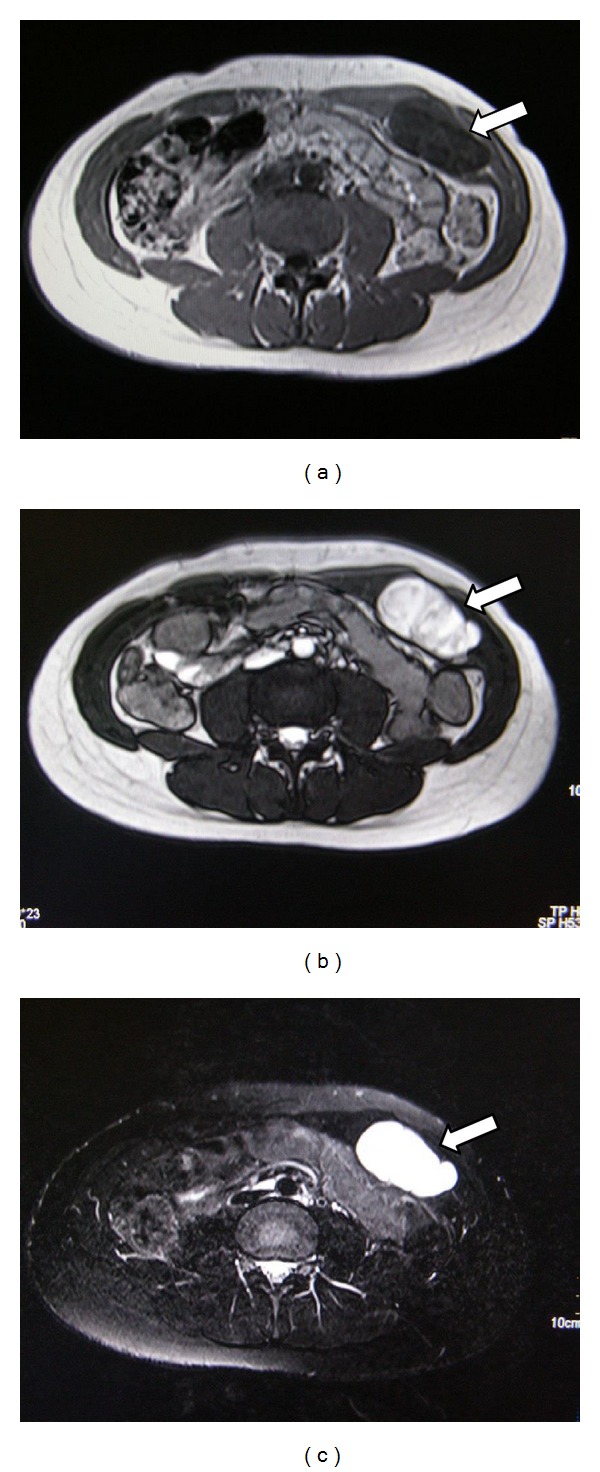
(a) T1W MRI shows encapsulated superficial mass with marked heterogeneity and patchy hypointense foci (arrow) suggesting cystic degeneration. (b) T2W MRI clearly delineates the capsule of the lesion and internal morphology (arrow). (c) The lesion is markedly hyperintense on fat-suppressed MRI suggesting degeneration (arrow).

Magnetic resonance imaging was performed which showed a solid, heterogeneous mass arising in the abdominal wall. It was hypointense on T1 and heterogeneously hyperintense on T2 and fat suppressed sequences suggesting cystic degeneration (Figures 2(a), 2(b), and 2(c)). A radiological diagnosis of neurofibroma was made. 

**Figure 3:**
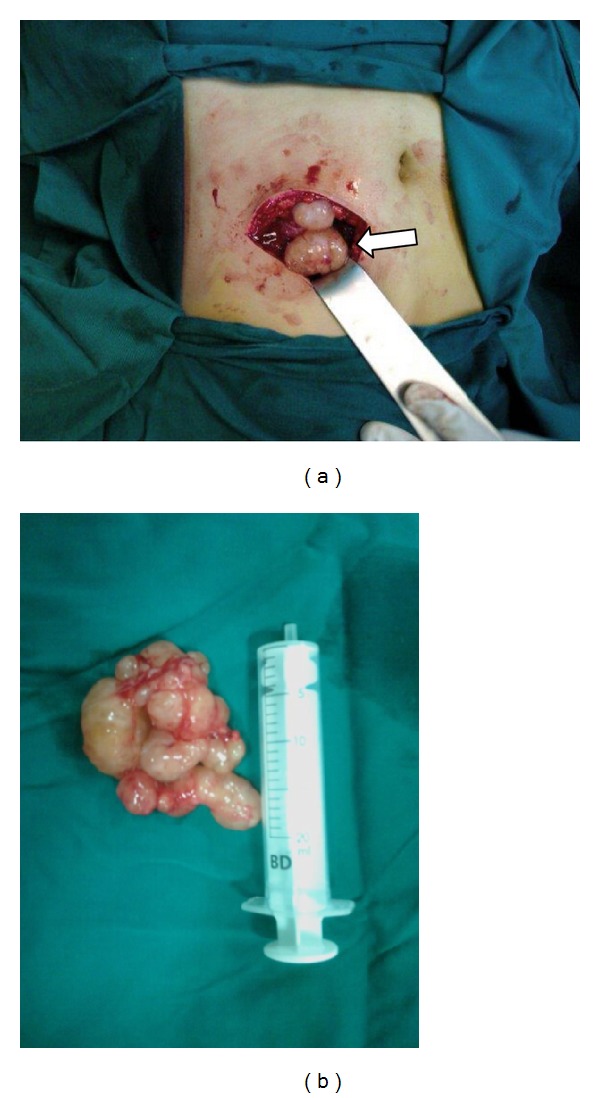
(a) Peroperative picture shows the superficial location of the mass (arrow). (b) Excised tumor.

**Figure 4:**
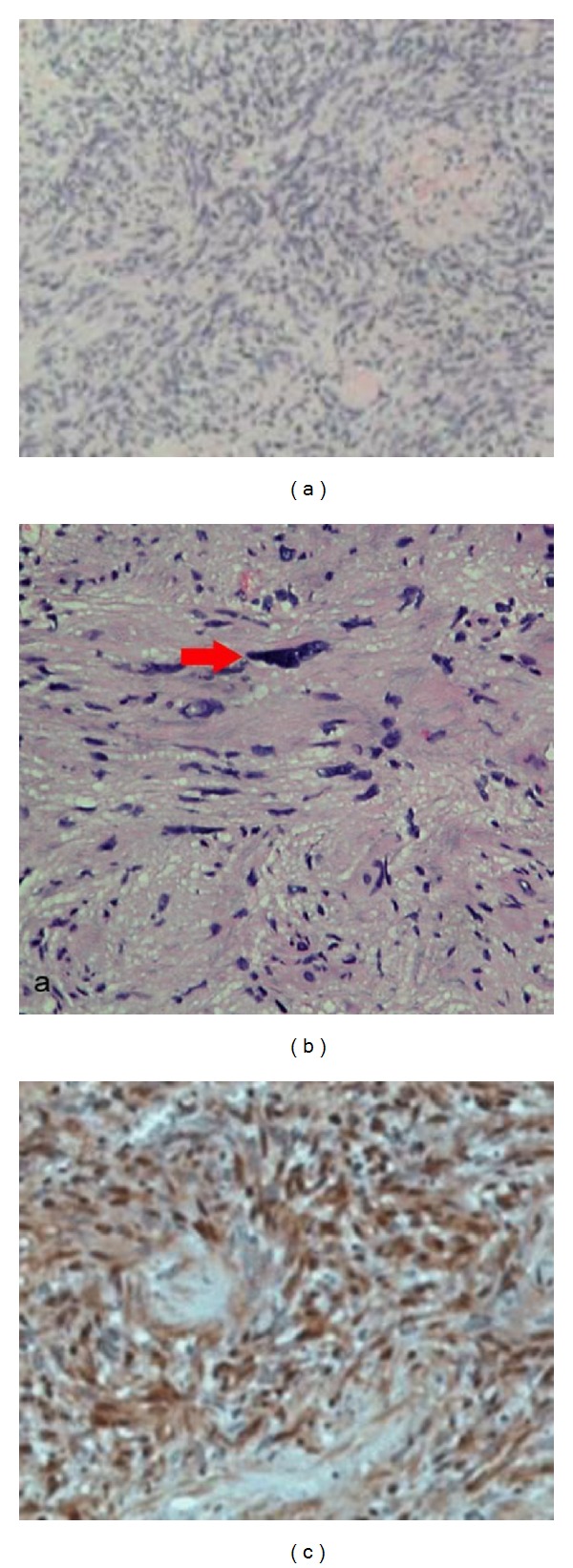
(a) Microscopic appearance of tumour showing Antoni A pattern with nuclear palisading (H & E staining). (b) Spindle cells in an Antoni type A area; red arrow indicates a large bizarre hyperchromatic nucleus. (c) Immunohistology slide confirming the diagnosis of schwannoma.

The tumor was completely resected en bloc and sent for histopathology. Macroscopically, the tumor was lobulated and pale yellow in color. (Figures 3(a) and 3(b)). Microscopically, it was composed of hypocellular and hypercellular areas. There was nuclear palisading (Figures 4(a) and 4(b)) although no mitotic figures were seen. Immunohistochemistry showed that the spindle cells were strongly positive for S100 protein (Figure 4(c)) and a final diagnosis of benign “Ancient” schwannoma was made. No evidence of malignancy or dysplasia was seen. 

The patient was discharged in good general condition and is now asymptomatic. 

## 3. Discussion

Ancient schwannoma is a rare ectodermal neoplasm arising from the nerve sheath that encases axons. Neurofibromas and schwannomas are benign peripheral nerve sheath tumors that occur as isolated sporadic lesions but have their major clinical impact on the neurocutaneous diseases, neurofibromatoses I and II. Schwannomas are also known as neurilemmoma. These occur most often in adult females [2] and are usually solitary. Up to 20% of cases are associated with neurofibromatosis type 1 [3]. They usually have predilection for head, neck, and flexor surface of extremities. However, there have been sporadic cases of these tumours arising in the porta hepatis [4], retroperitoneum, pelvis, adrenals, kidneys, and vagina [5]. Origin in the intercostal nerves can sometimes mimic a liver tumor [6] or a malignant tumor [7].

Most schwannomas arise from the nerve sheath of large peripheral nerves and occur below or at the level of the subcutaneous fat layer, even when they appear superficially. In the skin, schwannomas generally do not interfere with nerve conduction, but when they become large, they can compress the nerve of origin causing pain or dysaesthesia [8].

Considering the lack of symptoms and the superficial location of the schwannoma in our patient, it may have derived from a terminal cutaneous nerve. We suggest that the cystic degeneration may have contributed to a gradual increase in size of the tumor. 

Ancient schwannomas are a subtype of classic schwannomas with a predominance of degenerative changes including cyst formation, calcification, hemosiderin deposition, interstitial fibrosis, and vascular hyaline degeneration [9]. They show spindle cells with focal nuclear palisading patterns [3] arranged in distinctive dense (Antoni A) and loose (Antoni B) areas [3, 9]. The term “ancient” was used as a description for the degenerative changes apparent on microscopy [10, 11]. 

These lesions, on CT scan and MRI, appear homogenously solid while the “ancient” type has heterogeneous morphology. Schwannoma show intense enhancement with contrast while the “ancient” schwannoma would enhance nonuniformly but shows marked hyperintensity on T2 sequence characteristic of cystic degeneration as in our patient. 

Histologically, these lesions are well encapsulated, the capsule being derived from the nerve of origin. Schwannomas are composed of typical densely cellular areas (Antoni type A) alternating with myxoid, edematous areas (Antoni type B). As the Schwann cell proliferation is along the nerve, the nerve of origin is displaced to the periphery of tumor. Schwannomas react strongly with S100 protein and immunohistochemistry can be used to aid diagnosis and to differentiate them from malignant peripheral nerve sheath tumors [12]. Presence of hyperchromatic cells and atypia may cause suspicion for malignancy and immunohistochemistry in such situations is useful. 

The treatment of choice is surgical excision. Recurrence of schwannomas has been reported in the literature although some argue that malignant lesions may arise *de novo* [2]. It is possible that recurrence after excision may be due to incomplete resection [13]. Recurrence at a distant site has also been reported [14].

In this study we have presented the second reported case of an abdominal wall “ancient” schwannoma and discussed its clinical, radiological, and pathological features. This condition may not be as uncommon as yet considered.
